# High-Pressure Effects on Gelatin Sol–Gel Transition

**DOI:** 10.1021/acs.iecr.4c04861

**Published:** 2025-03-25

**Authors:** Nikolaos A. Burger, Gerhard Meier, Dimitris Vlassopoulos, Benoit Loppinet

**Affiliations:** † Foundation for Research & Technology Hellas (FORTH), Institute for Electronic Structure & Laser, Heraklion 70013, Greece; ‡ Department of Materials Science & Engineering, University of Crete, Heraklion 70013, Greece; § Biomacromolecular Systems and Processes (IBI-4), Forschungszentrum Jülich, 52425 Jülich, Germany

## Abstract

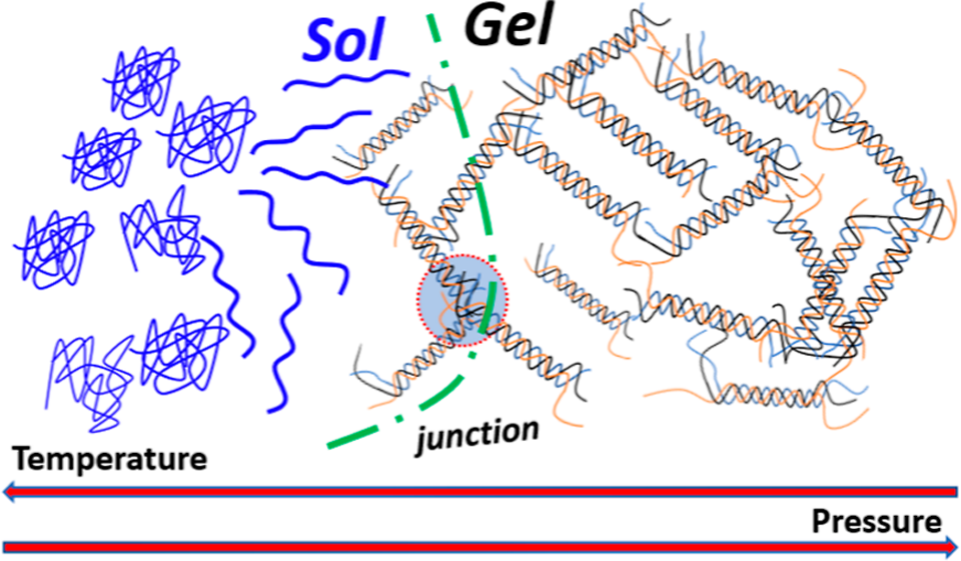

We investigated the effects of high hydrostatic pressure on the sol–gel transition of gelatin dispersions. We used dynamic light scattering (DLS) and DLS-based passive microrheology to monitor the evolution of the viscoelasticity during isothermal gelation. It provided easy identification of the sol–gel transition and the isothermal critical gelation time (*t*_c_) and values of viscosities of sols and shear modulus of gels. At a given temperature, *t*_c_ decreased with increasing pressure. Up to 100 MPa, the temperature dependence of *t*_c_ followed the established empirical rule  and the critical temperature *T*_c_ increased with pressure by ∼0.04 K/MPa. The critical gelation time scaled with the quench depth *T*–*T*_c_ or equivalently with the distance from the pressure-dependent collagen denaturation temperature (∼314 K, at 0.1 MPa), which also increases by ∼0.04 K/MPa in the first 100 MPa. The pressure dependence also reflected on the time evolution of the intrinsic viscosity, η_*i*_, or elastic modulus, *G*_p_, in the sol or gel state, respectively, are reported. Both η_*i*_ or *G*_P_ evolution speeds up with pressure. Finally, using a reverse quenching approach, we observed a slowing of the gel melting when the pressure increases. Our results confirmed that the rheological evolution reflects the helix formation process and that pressure stabilizes the helices.

## 1 Introduction

Gelatin is a material derived from the hydrolysis of collagen, the main protein component of connective tissues. It is considered as denaturated form of collagen and maintains the ability to form triple helix that is at the origin of its gelling properties.^1^ The use of gelatin is very widespread, from the model system for statistical physics, to cell growth medium, to foodstuff, and ingredients for glue or photographic films.^2−7^ When dissolved in water or alcohols, gelatin solutions have the ability to form gels that are the archetype thermo-reversible hydrogels.^8−12^ The gelation process has been widely studied and is now well understood. Above a temperature of approximately 38 °C (corresponding to the coil–helix transition observed in collagen), the gelatin macromolecules dispersed in an aqueous medium adopt a coil configuration. At high temperatures, the dispersions are always in a sol state independent of the gelatin concentration, though the sol viscosity increases gradually with the gelatin concentration and molecular weight.^13,14^ Below a critical temperature, helices (and in particular triple helices) start to form strong physical cross-links between different gelatin coils. This is a kinetic process, and as more cross-links form, the dispersion’s viscosity increases until the percolation point where a network of cross-linked gelatin coil spans the entire sample and the solution forms a macroscopic gel. The isothermal process can be well described by a percolation model with specific power-law dependence of the mechanical properties close to the percolation transition.^15−17^ In the gel state, as more helices continue to form, the gels exhibit an increase in the gel modulus with time, over a very long period of time. The slow kinetics (aging) of the well-matured gels give rise to a rich phenomenology that bears similarities with other arrested phases.^18^ The coil–helix transformation that drives the gelation process has been monitored using various techniques, such as optical rotation (OR), dialysis membrane, nuclear magnetic resonance (NMR), and differential scanning calorimetry (DSC).^19−22^ The consensus is that the helix formation is a nucleated kinetic process driven by specific type of hydrogen bonding with a specific transition temperature.^23,24^ An interesting relation between the helix content (measured with OR) and the modulus in the gel has been experimentally demonstrated.^13,17,25−27^ At ambient pressure, the sol–gel kinetics itself has been studied mostly through rheology, ultrasonics, or calorimetry.^14,24,26,28−35^ The influence of various parameters affecting the gelation (temperature and concentration but also pH, ionic strength, molecular weight, solvent environment, gelatin origin, and preparation) on its kinetics has been evaluated.^34−39^ The effect of high hydrostatic pressure (HHP) has comparatively been less investigated. Despite potential practical interest in food products, pharmaceutics, and processing, the influence of HHP on gelation of various biopolymers has received only limited attention.^40−49^ In particular, a HHP cycle has been proposed in food processing, as an alternative to temperature treatment for pasteurization and conservation.^50^ More relevant to the present work, the stability of collagen under HHP has been studied as a particular case of effect of HHP on thermal denaturation of protein.^51^ Falling ball viscosimetry, DSC, and NMR have also been used in the past to assess for the mechanism of gelation.^38,52^

Given the above, several open questions on how pressure influences the gelation remain. We discuss in particular the effects of HHP on the sol gel process and its kinetics. We monitor sol–sol, sol–gel, and gel–gel kinetics under isothermal (fast cooling to the final temperature) and temperature ramp over a range of pressure up to 100 MPa, for different gelatin concentrations. We employ light scattering-based passive microrheology and in situ dynamic light scattering (DLS) that are well suited for our HHP cells.^53−57^ We identify the critical gel point (and the associated time) through the ergodic to nonergodic transition (DLS) and the sol–gel transition (microrheology). We rationalize our findings in terms of the pressure dependence of thermodynamics and kinetics of the helix’s formation.

## 2 Materials and Methods

### 2.1 Materials

Gelatin powder with a bloom number of 300 purchased from Sigma-Aldrich was used. It has an estimated average molecular weight ∼10^6^g/mol.^9^ Solutions were prepared by dispersing the powder at room temperature in Milli-Q water and heating at 60 °C for 45 min under stirring, followed by a resting period of at least 24 h at room temperature. Three concentrations 2, 4, and 8% by weight were investigated. The rheological properties of gelatin dispersions are insensitive to pH changes (ranging between 4.6 and 8).^58,59^ No efforts to control the pH were made, and it was measured at ∼5.5 using pH paper. For microrheology experiments, we used as probes polystyrene (PS) latex particles with a diameter *d* = 191 nm (measured in the dilute regime by DLS) and nominal refractive index *n* = 1.598 (at 532 nm). They were purchased from Polymer Laboratories, Varian Inc. and used as received.^60^

### 2.2 Methods

#### 2.2.1 DLS Set-up

We used DLS to obtain the intermediate scattering function, *C*(*q*,*t*) of the gelatin dispersion. It allows one to monitor the sol–gel transition at different sample conditions (pressure, temperature, and concentration). DLS was also used to perform colloidal probe passive microrheology in both the single scattering DLS limit and the limit of diffusing wave spectroscopy (DWS) (Figure S1). Different light scattering setups were used, all including a monomode CW 532 nm laser as the light source (power of 80 mW). The scattered light was collected by a monomode optical fiber feeding two independent PMT in the photon counting mode and the cross correlations were computed by a hardware correlator (ALV6000). The time-dependent autocorrelation function was acquired for time less than 1 min to limit the evolution of the sample during that period. The acquired correlation functions were appropriate for analysis as described in the next paragraph. For the probe microrheology measurement, this short time allowed the retrieval of probe mean square displacement (MSD) with good statistics, while the samples remained in a quasi-steady state.^61^ Two different high-pressure light scattering cells were used, one using N_2_ gas, and one using hydraulic oil as a pressure transmitted medium.^53^ The N_2_ cell was used for measurements in the single scattering limit at 90° scattering angle for probe-free gelatin dispersion and also for single scattering in the presence of colloidal probe.^53,56,57^ The oil cell was used in transmission geometry with 2 mm thick samples for DWS (Figure S2).^55^ An analyzer was placed between the cell and the detector as a cutoff for polarized light (scattered or transmitted). Multispeckle detection using a CMOS camera as a detector and a software correlator^62^ were used to obtain the slow dynamics (as shown in Figure 2C) simultaneously with the fast dynamics captured by the PMT detection.^63^ The simultaneous detection was achieved using a beam splitter placed in the back of the analyzer for the transmission oil cell and two opposite 90° angle windows in the gas cell. For both cells, temperature control was achieved using a water circulation bath in the range from 10 to 70 °C. The pressure range was from 0.1 to 100 MPa. The refractive index of PS (1.598) compared to gelatin (1.4) and water (1.33) provided for large scattering from the probe. For single scattering DLS measurements, a probe volume fraction on the order of 10^–4^ was used. This value ensured that the probe scattering dominates the signal (>50x solution scattering) but remained in the single scattering regime. To achieve the turbidity required for DWS, a probe volume fraction of 0.5 wt % was used. The mean scattering path value was estimated using a Mie scattering calculator to be l* = 0.4 mm.^64^ Its ratio to the cell thickness *L*, *L*/l* = 5, is large enough to ensure DWS conditions.^65,66^ Further confirmation for the good estimate of the l* value was provided by the coincidence of the DLS and DWS MSD seen at high frequencies.

#### 2.2.2 DLS Analysis

We briefly present the analysis used in the different scattering experiments. The intensity correlation functions *g*_2_(*t*) delivered by the correlator is
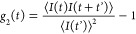
1where < > denotes average over the acquisition time. The intermediated scattering function or equivalently the field autocorrelation function (FAF), *g*_1_(*t*) ∼ *C*(*t*) is obtained using the Siegert relation, applicable for ergodic signals, is
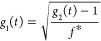
2where f* is an experimental factor (close to 1 in our case owing to the use of a monomode optical fiber) that characterizes the overall coherence and depends on the specifics of the illumination and detection used in the setup. In the case of nonergodic samples (here the gel samples), we considered the signal to be composed of a static component (scattering by a frozen network) and a fluctuating component (some motion present in the frozen network). The situation is identical to partial heterodyne conditions where frozen dynamics act as a nonfluctuating partial heterodyne light source and a generalized Siegert relation can be applied, with the normalized intensity autocorrelation now
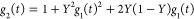
3with *Y* = *I*_E_/*I*_T_, the ratio of the ensemble *I*_E_ and the time average *I*_T_ intensities, respectively.^67^

Eq 3 was in particular used to rescale the PMT measured correlation function to the multispeckle camera-based correlation function.

In the case of colloidal probe microrheology, for both single scattering and transmission DWS, the probe MSD relates to the FAF (assuming that the scattering arises only from the particles) as
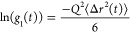
4where, the MSD is Δ*r*^2^(*t*) = *r*^2^(*t*)–*r*^2^(0), which is the displacement of the particle at lag time *t*. In the case of Brownian diffusion of the probe, the MSD is described by ⟨Δ*r*^2^(*t*)⟩ = 6*Dt*, with *D*, the diffusion coefficient. *Q* defines the inverse characteristic length scale of the technique. In the single scattering limit, *Q* is the scattering wavevector *q*.  where, *n* is the refractive index of the medium, , λ the wavelength in vacuum and θ is the scattering angle. In the case of transmission DWS, to a good approximation, *Q*^2^ can be expressed as
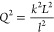
5In transmission DWS, the exponential decay relation between the MSD and correlation function assumed in eq 4 is an approximation of a more complex expression, known to be good approximation.^68−72^ Passive microrheology is based on the assumption that the Brownian motion of a large enough probe particle directly probes the linear viscoelastic spectrum of the material, expressed as the generalized Stokes–Einstein relation.^73,74^
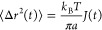
6where *J*(*t*) is the creep compliance of the material.

The frequency-dependent complex shear modulus defined as
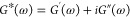
7with *G*′ and *G*″, the respective real and imaginary parts, were obtained by regularized Fourier transform of *J*(*t*) using the NLREG procedure as described elsewhere.^75−77^ As the transformation is an ill-posed problem, it inevitably introduces artifacts.^78^

The dynamic viscosity defined as
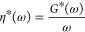
8was used in the case of sol. When extrapolated to zero frequency, it provided zero-shear viscosity.

Proper implementation of colloidal probe passive microrheology requires several conditions to be met: the measured scattering has to be dominated by the probe (low contribution of the sample itself, <2%), the probes have to be diluted enough to ensure that the motion of two separate probes is uncorrelated, and the probe size should be larger than any other length scale in the systems.^74^ It is widely assumed to be valid for both gelatin sol and gel dispersions.^79^

#### 2.2.3 Temperature and Pressure Protocol

The two different high-pressure sample cells imposed specific temperature protocols.

##### 2.2.3.1 Gas Pressure Cell

**Figure 1:**
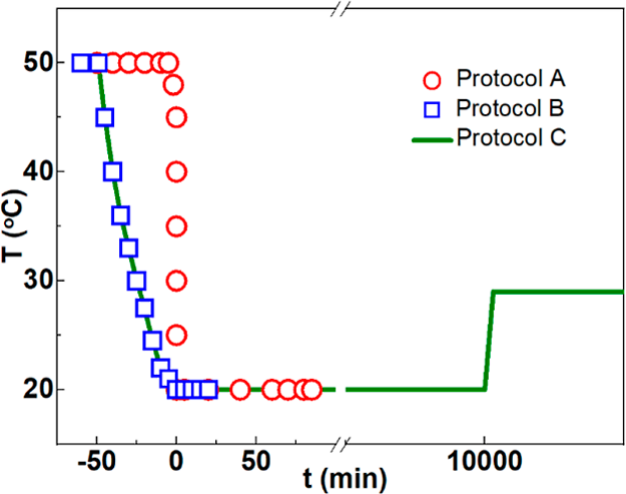
Different temperature treatments corresponding to different protocols: gelling procedure with the gas N2 cell (T-jump “isothermal” protocol A) and in the oil cell (temperature ramp protocol B); in the latter case, the cooling rate was ∼ 1 °C/min (from 50 to 20 °C) and inverse quenching treatment performed in the oil cell (protocol C) with cooling/heating rate ∼ 1 °C/min.

For fast quench, the gelatin dispersions were placed in a glass tubular light scattering cell and then heated at 50 °C for 10 min outside the HHP enclosure. The tube was then placed in the HHP cell set at the quench reference temperature (*T* = *T*_ref_). The large metal mass of the HHP cell and the low volume of sample ensured fast heat transfer and fast equilibration of the temperature from 50 °C to *T*_ref_. The HHP cell was then closed and the pressure was increased through compression of N2 reaching the set value in less than 5 min (protocol A in Figure 1A).

##### 2.2.3.2 Liquid Pressure Cell

The liquid cell design imposes some time delay between sample loading in the inner sandwich-pill (Figure S2) and its insertion in the HHP mount. Dispersions were first loaded in the sol state and the sandwich cell was mounted in the HHP mount preheated at 50 °C. The HHP mount was then closed and the pressure was set to the desired values. The HHP was then brought to the desired temperature with cooling/heating rates of the order of 1 °C/min through the use of a water circulation bath (protocol B in Figure 1). Inverse quenching experiments were also used (protocol C in Figure 1).^37^ The samples were first cooled from 50 to 20 °C as in protocol B and then kept for different waiting times at 20 °C and were heated to 29 °C and kept at this temperature for a long time (∼hours).

## 3 Results and Discussion

### 3.1 Detection of Sol and Gel Phases by DLS

**Figure 2:**
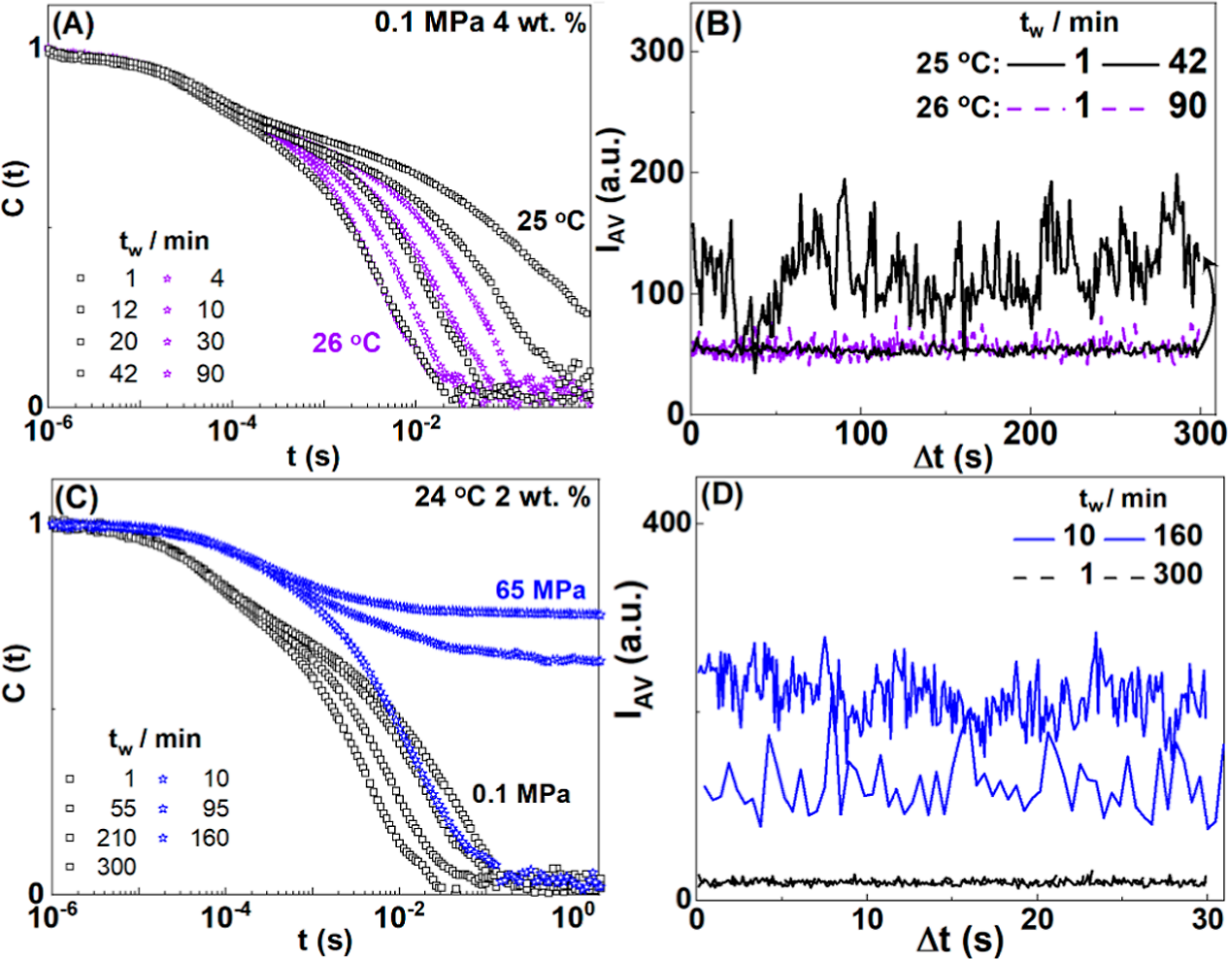
Identification of the sol–gel transition through DLS measurements: time evolution of *C*(*t*) of gelatin dispersions at (A) 4 wt %, 0.1 MPa, 25 °C (black squares), and 26 °C (purple stars) and (C) 24 °C, c = 2 wt %, 0.1 MPa (black squares), and 65 MPa (blue stars). Scattering intensity variations as a function of waiting time at (B) 0.1 MPa, 25 °C (black lines), and 26 °C (purple dashed lines) and (D) at 24 °C, 0.1 MPa (black dashed lines), and 65 MPa (blue lines). Data have been obtained after a temperature jump (50 °C to *T*_ref_ protocol A).

Shibayama and co-workers have shown how to detect the “critical” sol–gel transition using DLS and have established its coincidence with the rheological gelation.^80^ At the sol–gel transition, the intermediate scattering function, *C*(*q*,*t*), reflects the ergodic–nonergodic transition and loses its zero baseline. We here use the emergence of a power-law behavior in the *C*(*q*,*t*) region and the accompanying increase of the scattering intensity as a signature for the sol–gel transition. Typical correlation functions acquired during the isothermal gelation process are shown in Figure 2. Figure 2A shows the evolution of *C*(*q*,*t*) with the waiting time, *t*_w_, during the gelation process at ambient pressure (0.1 MPa) of 4 wt % dispersions at two different temperature quenches from 50 to 25 °C (black symbols) and 26 °C (purple symbols). One observes slow-down of the decay function, then the typical power law correlation, and then the emergence of a long time plateau.^81^ In sol conditions, the samples remained ergodic and the time average, (*I*_*t*_), is equal to the ensemble average, (*I*_E_), and *C*(*q*,*t*) have a long time baseline *C*(*q*,*t*) → 0^80^ with two well-separated relaxation modes, as observed in semidilute gelatin dispersions.^61^ The scattering intensity has a strong *q*-dependence with a power law *I∼q*^-2^ (Figure S3). With increasing *t*_w_, the slow relaxation process is shifted to longer relaxation times until eventually the correlation function becomes nonergodic. From the analysis of the *C*(*q*,*t*), the two characteristic decay rates were found *q*^2^–dependent, indicating diffusive character of the probed dynamics. The average intensity, *I*_AV_, was observed to increase with *t*_w_ as a consequence of the helix formation. We took the onset of nonergodic correlation as a measure of the critical gelation time (*t*_c_), which we discuss in Section 3.2 below. In the gel state, the sample becomes nonergodic and the time-averaged scattered intensity at a given q depends on the location of the probed volume in the sample.^80^Figure 2C,D compares isothermal gelation process of a 2 wt % gelatin dispersion following a temperature quench from 50 to 24 °C at 0.1 (black squares) and 65 MPa (blue stars), respectively. At higher pressures, the gelation proceeded much faster with a faster slowdown of the *C*(*q*,*t*) and faster evolution of the scattering intensity. Noticeably, at ambient pressure (black curves), the same dispersions remained in the sol state (ergodic correlation function) even after a waiting time of 300 min. More data of the evolution following the temperature quenches at different pressures are shown in Figure S4.

### 3.2 Gelation Time and Critical Gelation Temperature

**Figure 3:**
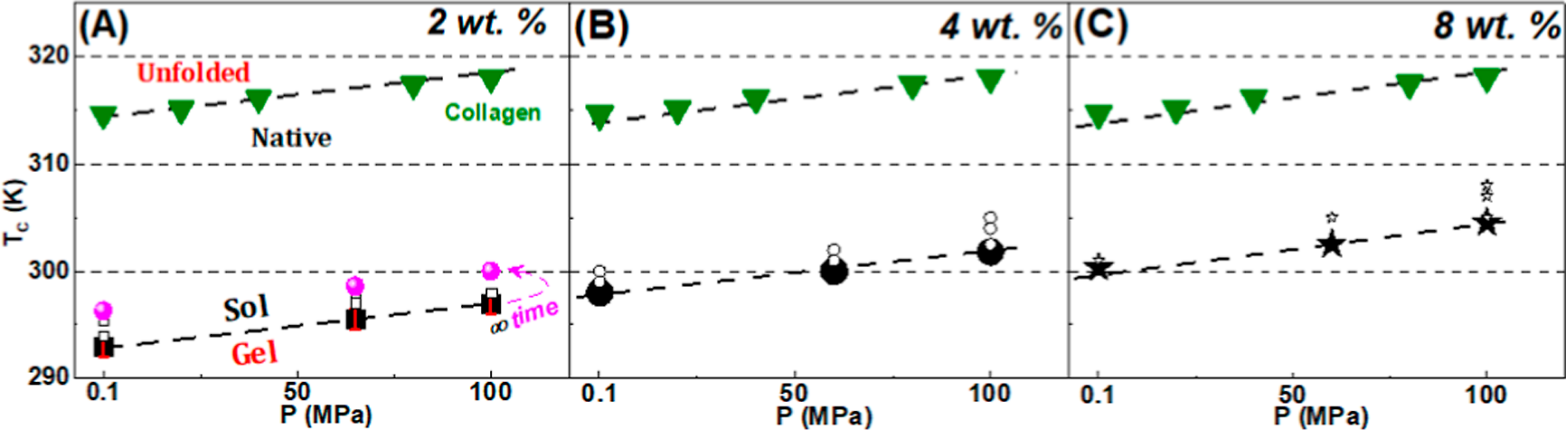
Sol–gel state diagram in the temperature–pressure space at different gelatin concentrations at: (A) 2, (B) 4, and (C) 8 wt %. Open symbols indicate sol state and filled symbols indicate gel state (squares, circles and stars, respectively). Points correspond to the observed state after 60 min, for the gel state and up to 300 min for sol states. Magenta circles indicate Tc as obtained from eq 8. Green triangles indicate unfolded to native state transition in collagen taken from ref (51). The dashed lines indicate a slope of 0.04K/MPa. Data have been obtained after a temperature jump (50 °C to *T*_ref_ protocol A) .

In Figure 3, we clearly see the increase in the sol–gel transition temperatures with increasing pressure. Interestingly, the sol–gel temperature lines are parallel to the helix–coil temperature ones, our pressure-dependent shift of the sol–gel transition is similar to the 0.04 K/MPa shift of the coil–helix transition in the collagen. This holds for a broad range of concentrations (Figure 3A–C). It is worth pointing out that both gelation temperatures *T*_1/h_ (black squares) or *T*_c_ (magenta spheres) display similar 0.04 K/MPa pressure dependency. This data also appeared to be in good agreement with the study (through falling ball test) of Gekko and Fukamizu^44^ who reported the stabilization of the gel phase with increasing pressure and attributed it to the change of the molecular volume at higher pressures.

**Figure 4:**
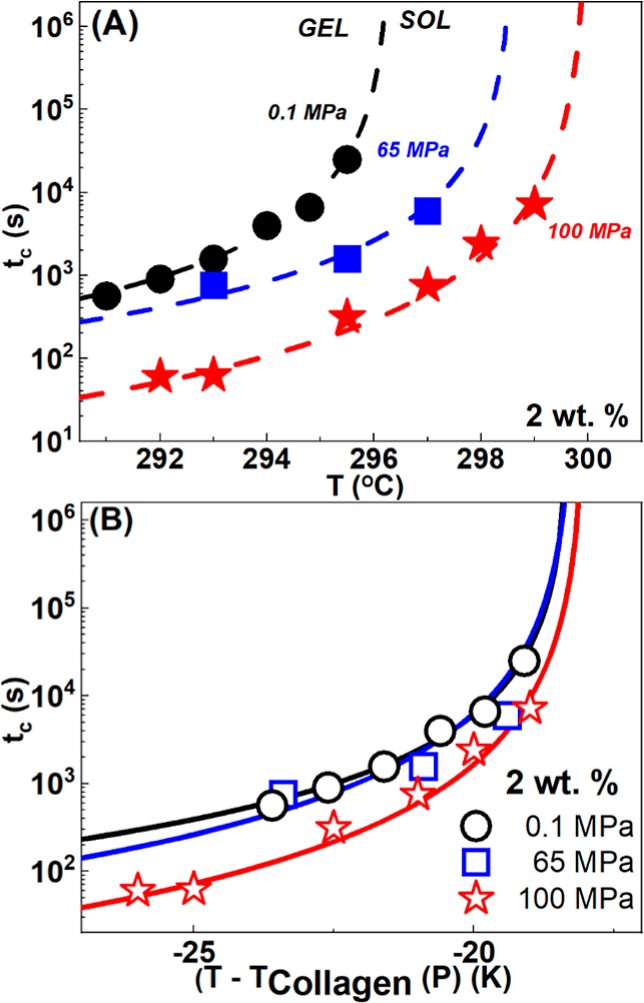
(A) Critical gelation time (*t*_c_) for c = 2 wt % as a function of quench temperature at different pressures at 0.1 (black circles), 65 (blue squares), and 100 MPa (red stars). (B) Same data as in (A) with the temperature axis rescaled with the respective collagen denaturation. Lines in (A) and (B) are fits of the experimental data to eq 8. Data have been obtained after a temperature jump (50 °C to *T*_ref_ protocol A).

A complementary way to assess the effect of pressure is to consider the sol–gel transition as a kinetic process and, therefore, to use the critical gelation time, *t*_c_, to quantify it. It is defined as the time that it takes to reach the critical gelation following temperature quenches. The critical gelation time, (*t*_c_), is plotted in Figure 4A as a function of the quench temperature for the 3 pressures.

We observe that *t*_c_ drops significantly with increasing pressure so that at a given temperature gelation proceeds faster as pressure is increased. The dependence of *t*_c_ on the different parameters is often described by the empirical equation first proposed by Ross-Murphy.^82,83^
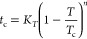
9where *T*_c_ is the critical temperature, corresponding to the highest gelling temperature where gel formation would take infinite time, *K*_*T*_, (with units of time) corresponds to the fastest gelation time that would be observed in case of deep quench (*T* ≪ *T*_c_) and *n* is a critical exponent.*K_*T*_* and *n* are both concentration dependent.^82,83^ Values of *n*, *K*_*T*_, and *T*_c_ are treated as fit parameters.The values of the parameters obtained from fitting the pressure-dependent critical time temperature evolution are shown in Table 1 below. Given the few experimental points, the results need to be considered with care, but we observe that *T*_c_ bit also *K*_*T*_ and *n* depend on the pressure (Figure S5). Interestingly, the effect of pressure looks to be similar to that of concentration (Figure S6).

**Table 1:** Fit Parameters of eq 9 for the Experimental Data Shown in Figure 4

pressure (MPa)	*T*_c_ (K)	*K*_*T*_ (s)	*n* (−)
0.1	296.3	0.2	–1.98
65	298.6	0.2	–2.02
100	300	0.006	–2.43

The fits with eq 9 are shown as dotted lines. The same data are plotted (Figure 4B) vs the distance to the pressure-dependent collagen denaturation temperature.^51^ The sol–gel boundaries at different pressures almost superimpose, suggesting that the distance (in the temperature space) to the collagen denaturation temperature is the key parameter affecting the sol–gel kinetics. An increase of pressure seems to correspond to a deeper temperature quench. A small deviation at small *t*_c_ and small temperature differences is observed even after the normalization.

### 3.3 Sol Viscosity

**Figure 5:**
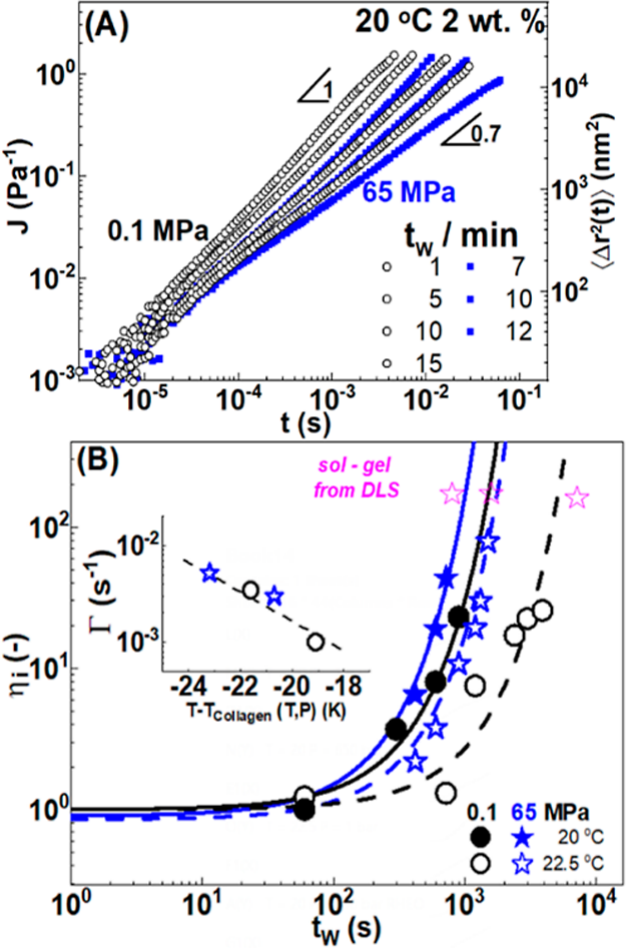
(A) Temporal evolution of the creep compliance (and the equivalent MSD on the right vertical-axis) of gelatin c = 2 wt % dispersions at 20 °C at 0.1 MPa (ambient pressure) (black circles) and 65 MPa (blue squares). (B) Intrinsic viscosity as a function of waiting time (*t*_w_) for different pressures and temperatures at 20 and 22.5 °C at 0.1 MPa (black circles) and 65 MPa (blue stars). Data were obtained after a temperature jump (50 °C to *T*_ref_) according to protocol B. Lines are fits of the data with η_*i*_ = *A* exp(Γ*t*_w_)(see text). Inset: the growth rate as a function of the temperature difference from the respective collagen denaturation temperature. The magenta open stars indicate to gelation time *t*_c_ obtained by DLS (shown in Figure 4).

The time dependence of the viscosity and the elastic modulus of gelatin dispersions has been discussed in the literature. An important outcome is the relationship between the elastic modulus and helix amount that has been proposed.^13,34^ However, the temporal evolution of the sol viscosity during the sol–gel transition is less documented.^13^ Following different temperature–pressure quenches, we observed a systematic evolution in colloidal probe DLS data of 2 wt % gelatin dispersions. Figure 5A shows the MSD (⟨Δ*r*^2^(*t*)⟩, right vertical-axis) of the colloidal probe, or equivalently the sample creep compliance at different waiting times (*t*_w_) and pressures of 0.1 (black circles) and 65 MPa (blue squares). The respective *C*(*t*) values are illustrated in Figure S7.

At 20 °C and 0.1 MPa (black symbols), the probes undergo diffusive motion and *J*(*t*) ∼ *t* for approximately 15 min before a subdiffusive motion is observed with *J*(*t*) ∼ *t*^0.7^. This reflects the onset of viscoelasticity. At 20 °C and 65 MPa, the probes undergo diffusive motion (*J*(*t*) ∼ *t*) only for *t*_w_ < 10 min. At *t*_w_ > 15 min, a subdiffusive motion is observed before *J*(*t*) substantially deviates from linear time dependence. At 22.5 °C and 0.1 MPa, the kinetic process became significantly slower (Figure S7). Figure 5B depicts the evolution of the intrinsic viscosity () with the waiting time *t*_w_.^84^ In this representation, η_*i*_ accounts for changes in solvent viscosity with the temperature and pressure. In the case of subdiffusive ⟨Δ*r*^2^(*t*)⟩, the viscosity values were obtained using an average diffusion coefficient deduced using CONTIN analyisis as detailed in the Supporting Information.^85^ The viscosity was deduced using Stokes–Einstein equation ().

We expect the viscosity to diverge at the gelation point. To empirically assess the temporal evolution, we used instead an exponential increase of η_*i*_ with *t*_w_, η_*i*_ = *A* exp(Γ*t*_w_) as it fitted well with our data, with *A*, a constant, and Γ, a growth rate in s^–1^. The inset in Figure 5B shows the evolution of the exponential growth rate (Γ) with the temperature, reported as the distance to collagen denaturation temperature (*T*–*T*_collagen_). Γ vs *T* is shown in Figure S11. The rates at different quench temperatures and pressures superpose well when plotted against the temperature difference from the collagen denaturation temperature (inset of Figure 5B). The distance to the denaturation temperature controls both the critical gelation time but also the temporal evolution of intrinsic viscosity. The evolution of the η_*i*_ is expected to be governed by the increased cross-link density as the helix formation proceeds. The universal rate dependence shown in the inset of Figure 5B provides an indication that the rate of helix formation is mostly driven by the quench depth (referred to the helix–coil transition temperature).

When passing from sol to gel, the MSD or equivalently the creep compliance *J*(*t*) adopted a power law behavior, as hinted in Figure 5 and also shown in Figure S8. This is equivalent to the Winter Chambon critical gel criteria.^88^ The self-similarity underlying the power law allows the determination of the critical gel point. A time–cure superposition is often used to allow precise evaluation of the criticality.^86,87^ As discussed above, we have used the onset of the power law in the pure gelatin dispersion intermediate scattering function here as a criterion for gelation. It is worth noting that both criteria lead to very similar values of the two critical gelation times *t*_c_, as shown in Figure S8.

The magenta stars in Figure 5 indicate the critical gelation time obtained from the DLS measurement and are shown in Figure 4. Clearly, we were not able to report viscosities in that time range. They would correspond to intrinsic viscosities larger than 100. A more precise analysis of the sol–gel transition and the nature of the dynamics in this region would be possible using the time–cure superposition^86,87^ but lies out of the scope of this work.

### 3.4 Gel Elasticity

**Figure 6:**
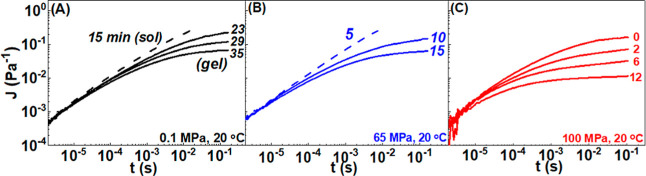
Time-dependent creep compliance *J*(*t*) of 2 wt % gelatin dispersions following temperature quench at 20 °C at: (A) 0.1 (black lines), (B) 65 (blue lines), and (C) 100 MPa. Measurements were performed according to protocol B.

During the sol–gel transition, the scattering intensity from the gelatin itself increased (see the inset in Figure 2) and eventually reached values comparable to the probe scattering. This makes the colloidal probe DLS approach ineffective. Hence, we used colloidal probe DWS microrheology to obtain the evolution of creep compliance *J*(*t*) with *t*_w_ in the gel state. Typical *J*(*t*) data measured a different waiting times are depicted in Figure 6 for sample cool down to 20 °C (protocol B) at different pressure at 0.1 (black), 65 (blue), and 100 (red lines) MPa. At short times (<10^–4^ s), a power-law dependence *J*(*t*) ∼ *t*^0.75^ is observed, as discussed above. Such a power-law exponent has also been reported for other gelling soft materials and attributed to the flexibility of the gel.^89^ We found that the longer the *t*_w_ (indicated with numbers in minutes in Figure 6), the smaller the creep compliance or the equivalent ⟨Δ*r*^2^(*t*)⟩ power-law exponent, indicating an increased elasticity. At the longest *t*_w_, the compliance reached a plateau value *J*_p_, from which the elastic shear modulus was extracted as *G*_p_=1/*J*_p_. We also show in Figure S9 the dynamic shear modulus obtained through the use of NLREG on *J*(*t*). Clearly, *G*_p_ corresponds to the *G*′ data at the lowest frequencies.

**Figure 7:**
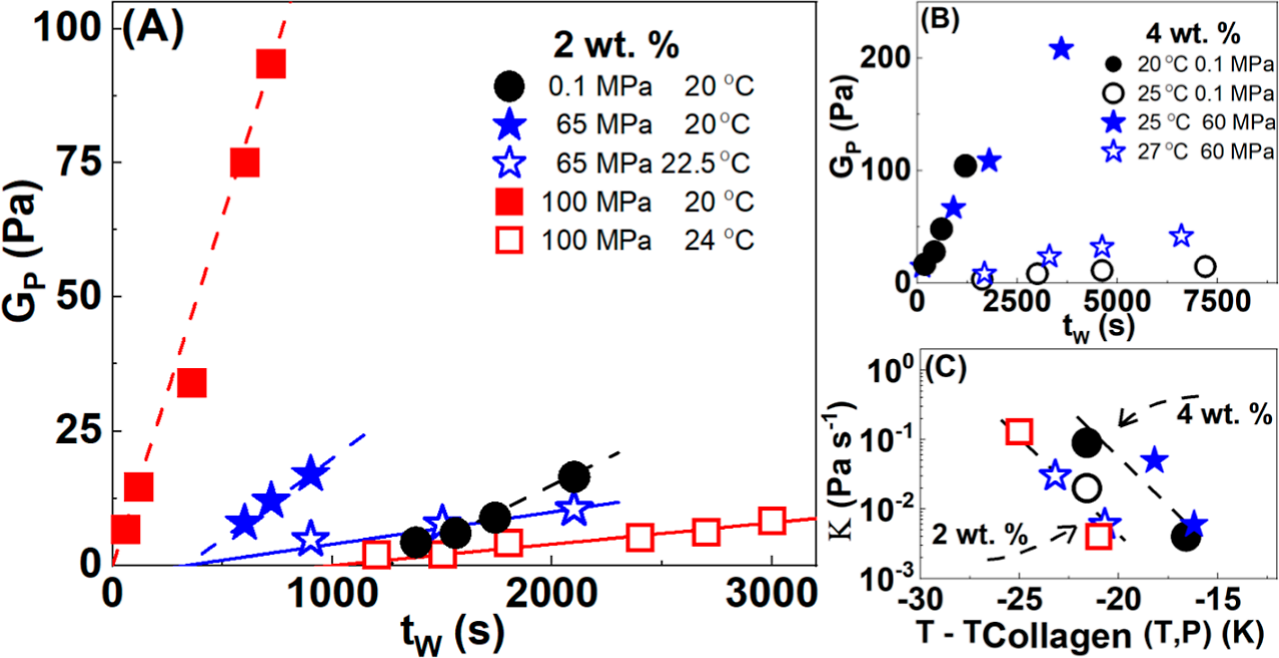
Evolution of the plateau modulus (*G*_P_) of gelatin dispersions following different temperature quenches at (A) 0.1 (black lines), 65 (blue lines), and 100 MPa (*c* = 2 wt %), and (B) 0.1 (black lines) and 60 (blue lines) MPa (*c* = 4 wt %). (C) Rate *K* extracted from the linear fit in (A) and (B). Measurements were performed according to protocol B.

In Figure 7, we show the evolution of plateau modulus *G*_p_ with waiting time *t*_w_ for different temperatures and pressures. Beyond the gelation point, we observe that the plateau modulus increases linearly with time *G*_p_ = *K*(*t*_w_–*t*_c_), where *K* is the rate of increase in the modulus. Data are reported at two different gelatin concentrations, 2 wt % (Figure 7A) and 4 wt % (Figure 7B), following different pressure and temperature quenches. At a constant quench temperature, the higher the applied pressure, the higher the rate of increase of *G*_p_. The evolution of rate *K* with the temperature difference is shown in Figure S11. Again, as for the growth rate extracted from the intrinsic viscosities, the rescaling appears to lead to the superposition of the different pressure measurements. The driving force for the rheology of the gelatin dispersions known to be the rate of helix formation seems to rescale well with the distance of the collagen denaturation temperature when the pressure is changed.

**Figure 8:**
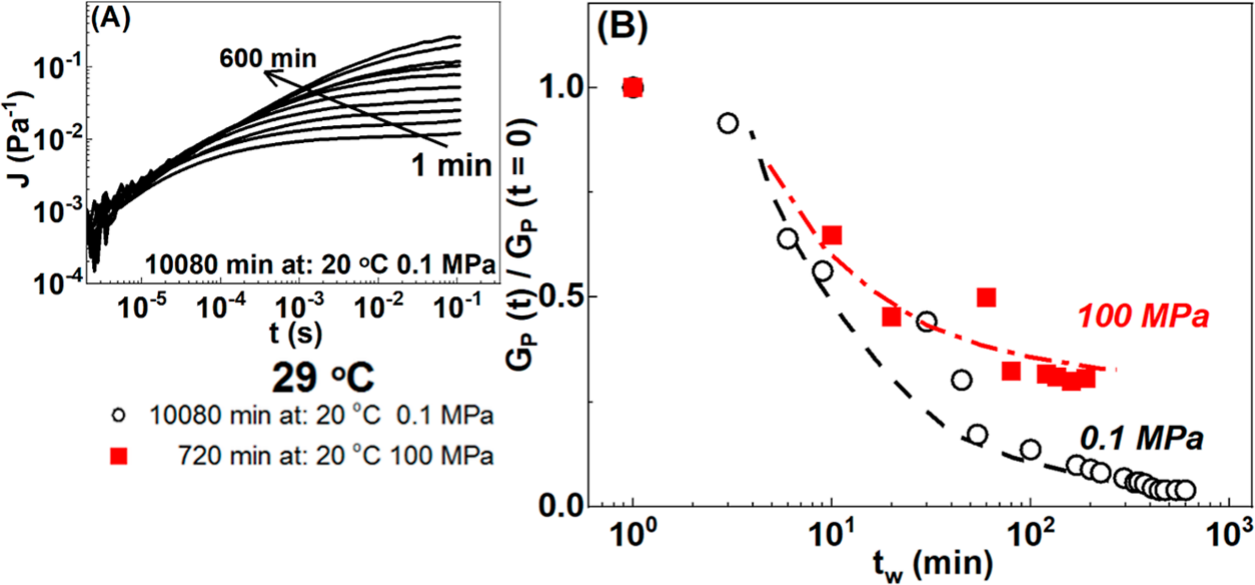
(A) Time-dependent on creep compliance, *J*(*t*) at 29 °C and different waiting times from 1 to 600 min (black solid lines), for dispersion prepared at 10,080 min, 0.1 MPa, and 20 °C. (B) Evolution of the normalized plateau modulus *G*_p_(*t*)/*G*_p_(0) with waiting time *t*_w_ at 29 °C following different gel preparation times or pressures for a 2 wt % gelatin solution. Corresponding *G*_p_(*t*) are shown in Figure S12. Reverse quenching measurements performed according to protocol C.

To further check the effect of pressure on the gels, we performed melting experiments also referred to as reverse quenching (protocol C in the Materials and Methods section). It has been proposed as a good protocol to follow rheological evolution during a kinetic process.^37^ For gelatin, it has been used for DSC experiments^21^ and also for rheology.^37^ Gels matured at a reference temperature were then brought to a higher temperature, and the isothermal evolution of the plateau modulus was followed, revealing first the softening and eventually the melting of the gels. We applied this protocol by introducing the effect of pressure. We followed the isothermal evolution (decrease) of the plateau modulus at 29 °C for different pressures. The temperature was picked up based on the results of Figure 4A, as it is in the sol regime for the investigated pressures. In Figure 8A, we report the creep compliances *J*(*t*) of a 2 wt % gelatin matured for 7 days at 20 °C, at different waiting times following the temperature increase to 29 °C. The slow evolution of the modulus during the maturation is shown in Figure S10. In Figure 8B, we report the time evolution of the plateau modulus, G_P_, obtained as 1/*J*(*t*) at large time and rescaled with the plateau modulus at *t*_w_ = 1 min. It is compared with a similar measurement for gel softening at 29 °C but at 100 MPa high pressure, after 12 h maturation at 20 °C and 100 MPa. The unscaled *G*_P_ vs *t*_w_ curves are shown in Figure S12 together with other samples. For the gel prepared at atmospheric pressure (open black symbols), the modulus drops significantly and continuously with time until almost melting, reflecting the decrease of helix contents upon remelting. At high pressures (100 MPa), the gel also softened but the modulus reached a saturation level as the ratio stabilized around 0.25 after ∼ 50 min^37^ Interestingly as shown in Figure S12, the ratio further decreased when the pressure was lowered from 100 to 0.1 MPa. The melting experiments further exemplified that the distance between the temperature and the helix–coil transition (quench depth) mostly controls the isothermal evolution. The 100 MPa pressure corresponds to a shift up of 4 K in critical temperature, so that a gel at that 29 °C can melt at ambient pressure, remaining gel at 100 MPa and further remelt when brought to ambient pressure. Our observation fits well with literature studies on the effect of pressure on the melting temperature.^44,51^

The sol to gel (or gel to sol) transition in gelatin dispersions is a consequence of the collagen triple helix formation (or melting). It provides an example of an aggregation kinetics and its coupling with the rheological properties. Upon cooling below a certain temperature, helices form and the dispersions are first in the sol state until the transition to a macroscopic gel state, with a characteristic elastic modulus. The modulus further increases as the helix formation proceeds in the gel state. The formation of helices is best monitored by OR.^13^ Rheology does not probe directly the rate of formation of triple helices but rather its consequences for the viscoelasticity. A generic relationship between the OR and the modulus has been put forward.^26,34^ Percolation theory has long been used to describe critical gelation in particular in polymeric systems, either chemical or physical.^16,20^ When a critical number of bonds per molecule is reached, the material undergoes a sol–gel transition where it changes from a viscoelastic liquid to a viscoelastic solid. At the gel point, the solution viscosity, the average molar mass, and the correlation length diverge. Macroscopic properties like intrinsic viscosity and modulus follow scaling laws close to the gel point and in particular, *G*(*t*)∼*t*^*u*^. For gelatin, careful studies have identified two gelling regimes, fast and slow gelling depending on the kinetics of the helix formation, where in the fast gelling regime *u* = 0.45.^13^ In this study, we focused on the kinetic evolution of the mechanical properties and the temporal concentration fluctuations (DLS) as we did not monitor the OR and, therefore, could not access the helix concentration directly. We focused on the effect of pressure on the gelation kinetics, which was well captured by our experiments. We conjectured that the main effect of pressure is the shift of *T*_c_ that follows the shift of the temperature of collagen helix melting. When it comes to the effect of pressure on the criticality, we did not observe any changes with an increasing pressure. We also did not see significant qualitative changes in the gelation kinetics. We, therefore, conclude that the same helix forms at different pressures; i.e., the gelation follows a similar path, and only the time dependence is affected by the pressure.

Our study further confirms that the quench temperature (quench depth *T*_c_–*T*) is the main parameter controlling the kinetics of helix formation at every pressure. The pressure effect is mostly on *T*_c_ with an increase of 0.04 K/MPa. At larger pressures, the same temperature results in a larger quench as *T*_c_ increases with pressure. Our measurements on the effect of pressure on gelation are also in agreement with the previously reported gelatin melting under high pressures with the less accurate falling ball method.^44^ The helix–coil transition of collagen (and in gelatin) is described as a nucleation and growth process. The pressure mostly affects the homogeneous nucleation rate (which is the rate-determining step) by lowering the energetic barrier.^25^ Quantitatively, our results are in good agreement with the DSC results of collagen.^51^ The speed-up of gelation is attributed to the change in activation energy of the collagen helix–coil transition, i.e., the change in activation volume in the protein helix formation. The similarity between the collagen denaturation and the gel melting or forming is taken as evidence that the activation volume is directly related to that in the collagen.

## 4 Conclusions

Using DLS and passive microrheology, we investigated the effects of pressure on the isothermal gelation kinetics of gelatin dispersions. Over the examined range of concentration, pressure, and temperature, the critical gelation time (*t*_c_) follows the empirical rule of Ross-Murphy, . Pressure affects both the characteristic time *K*_*T*_ and the critical temperature *T*_c_. *T*_c_ increased by 0.04 K/MPa, a very similar slope to the pressure dependence of the collagen helix to coil transition temperature. Passive microrheology allowed us to examine the evolution of the intrinsic viscosity (η_*i*_) in the sol state and the plateau modulus (*G*_P_) in the gel state, at different pressures. Both the evolution of η_*i*_ and *G*_P_ rescaled well with the quench depth *T*–*T*_c_.

Gels formed at a given time and at a given temperature were found to be stronger on increasing pressure. Similarly, dispersions prepared at high pressures can form a gel at higher temperatures. Most importantly, our work confirms that the kinetics is controlled by the quench depth, the distance between the set temperature and *T*_c_ at all pressures and that pressure stabilizes the helix formation.

## Supporting Information Available

Schematic illustrations of the methods and of the high-pressure oil cell; dynamic and static light scattering of gelatin dispersion at T,P,c; rescaled *t*_c_ vs T plot and evolution with P; plot of *t*_c_ vs T for all samples considered; description of the CONTIN analysis used to extract for the sol viscosities and examples of colloidal probe DLS correlogram and equivalent *J*(*t*); examples of power law in *J*(*t*) at the gel-point and comparison of *t*_c_ extracted from *J*(*t*) and DLS; *G*′(ω) obtained through NLREG transformation of *J*(*t*), rates of gel and sol evolution extracted from Figures 5B and 7C; evolution of *G*_P_ with time after reverse quenching (as in Figure 8B) (PDF)
