# Supplementary material for: High-Pressure Effects on Gelatin Sol–Gel Transition
Source: Ind Eng Chem Res. 2025 Mar 25;64(14):7370–80. doi: 10.1021/acs.iecr.4c04861 (PMC11987016; doi:10.1021/acs.iecr.4c04861)
Supplement: Supplementary file 1 — ie4c04861_si_001.pdf [file ie4c04861_si_001.pdf]

High pressure effects on gelatin sol-gel transition

Nikolaos A. Burger[1, 2], Gerhard Meier[3], Dimitris Vlassopoulos[1, 2], Benoit Loppinet[1*]

[1]*Foundation for Research & Technology Hellas (FORTH), Institute for Electronic Structure & Laser, Heraklion 70013, Greece*

[2]*University of Crete, Department of Materials Science & Engineering, Heraklion 70013, Greece*

[3]*Forschungszentrum Jülich, Biomacromolecular Systems and Processes (IBI-4), 52425 Jülich, Germany*

**E-mail: benoit@iesl.forth.gr*

Contents

i. Schematic illustration of methods and the experimental Set – up

ii. Gelatin dispersion DLS

iii. Gelation time and critical gelation temperature

iv. Sol viscosity

v. Gel elasticity

i. Schematic illustration of methods and the experimental Set – up

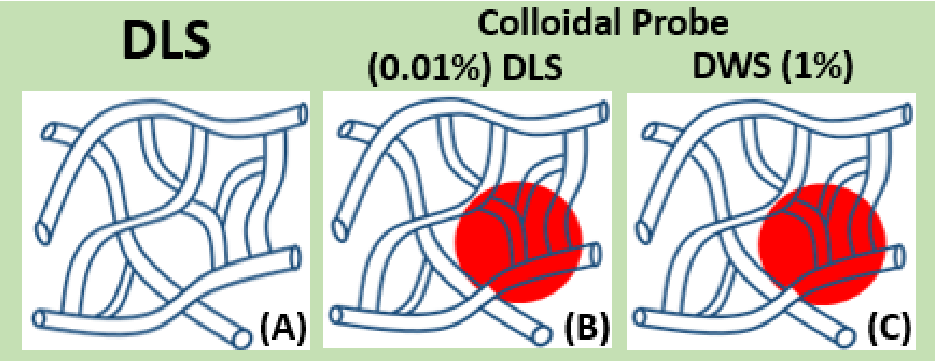

Figure S1. Summary of the three different experimental approaches useed in this study. Cartoons represent out of scale gelatin random coils (blue) and polystyrene probes (red spheres). (A) 'In – situ' DLS for the characterization of the sol-gel transition. (B) Colloidal probe single scattering DLS experiments with low fraction addition of polystyrene microspheres (~0.01 wt. %). (C) Colloidal probe DWS experiments with addition of ~0.5 wt. % polystyrene microspheres.

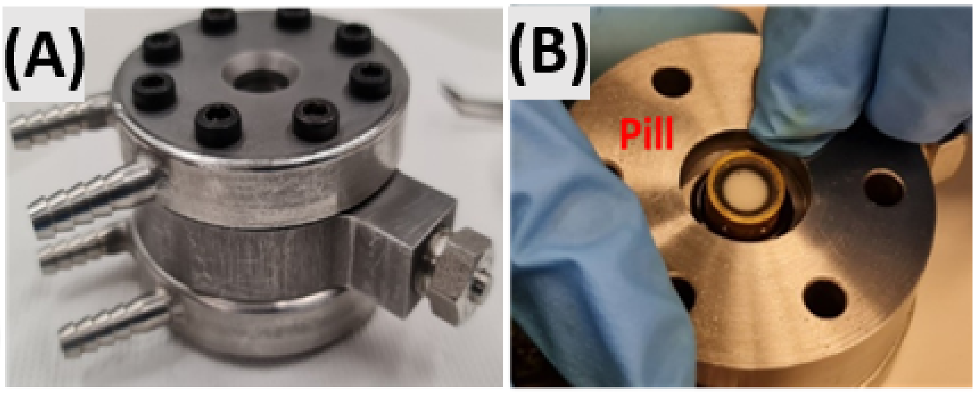

Figure S2. (A) High-pressure oil cell full view. (B) Inner view: a pill consisting of two sapphire windows separated by a Viton O-ring and enclosed in a brass ring is holding the sample. The pill placed the inner cell holder where it is sandwiched between two glass windows. The multiple scattering sample appears as milky. The black O-ring and the brass ring are clearly visible on the picture.

ii) Gelatin dispersion DLS

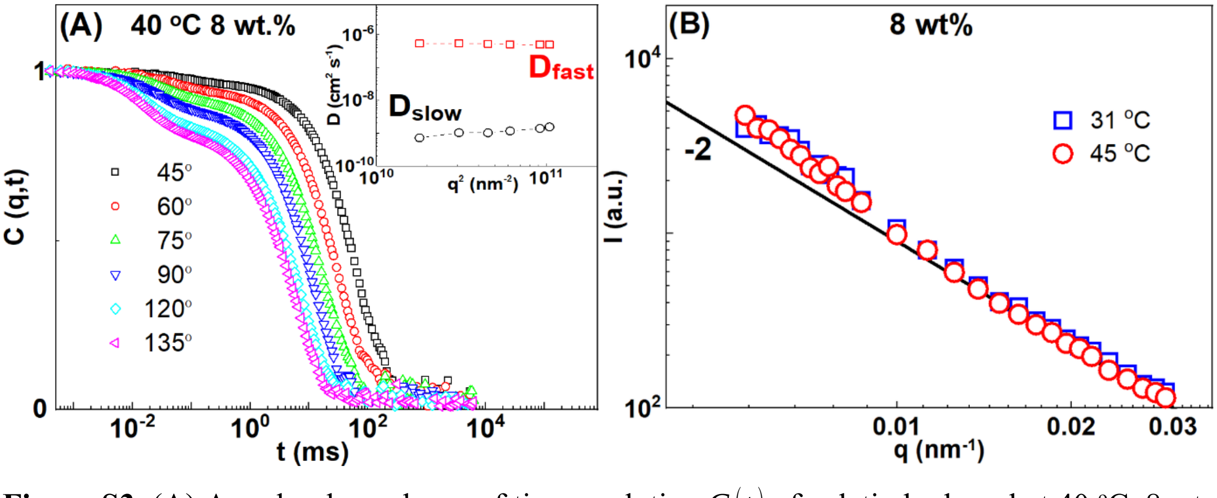

Figure S3. (A) Angular dependence of time evolution $C(t)$ of gelatin hydrogel at 40 ºC, 8 wt. %, 0.1 MPa. Inset: Respective fast and slow diffusion, (B) Scattering intensity as a function of wave vector (q) from the same sample at different temperatures, 0.1 MPa.

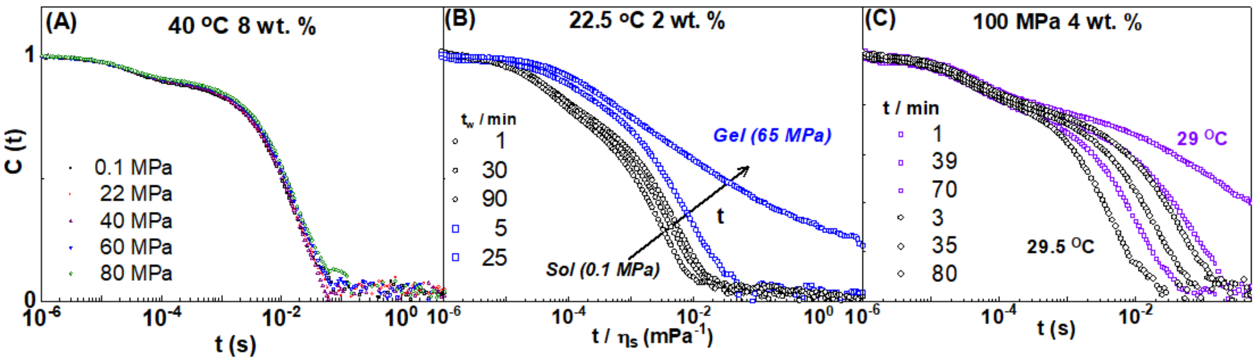

Figure S4. Time evolution $C(t)$ of gelatin dispersions at: (A) 40 ºC, 8 wt. % and different pressures, (B) c = 2 wt. %, 24 ºC, at 0.1 MPa (black circles) and 65 MPa (blue squares) and (C) c = 4 wt. %, 100 MPa at 29 ºC (purple squares) and 29.5 ºC (black circles).

iii. Critical gelation time and critical gelation temperature

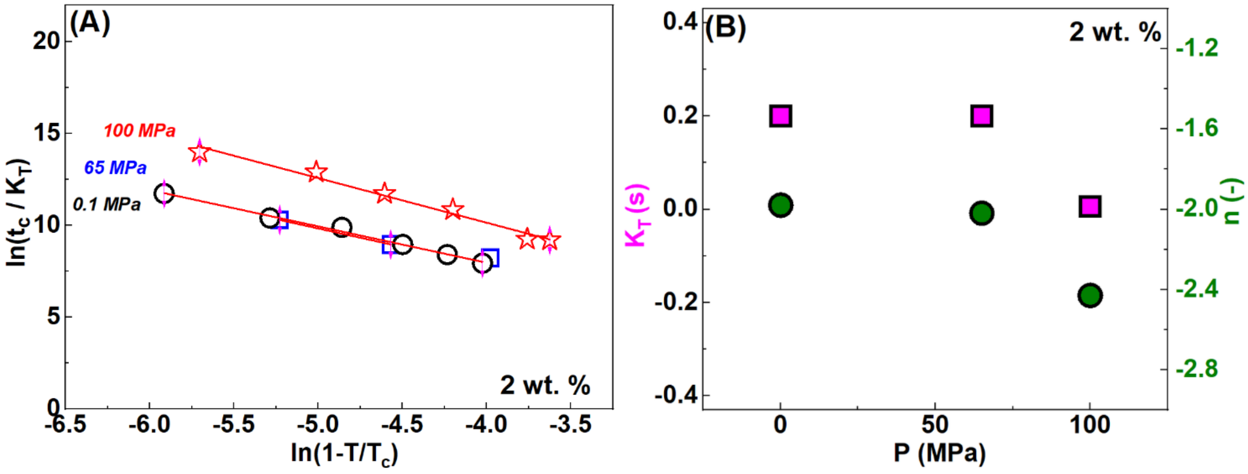

Figure S5. Dimensionless plot of the Ross-Murphy equation at 0.1 (black circles), 65 (blue squares) and 100 MPa (red stars) c = 2 wt. %. (B) K_T and n parameters as a function of pressure. Data extracted after Temperature jump (50 ºC to T_{ref}).

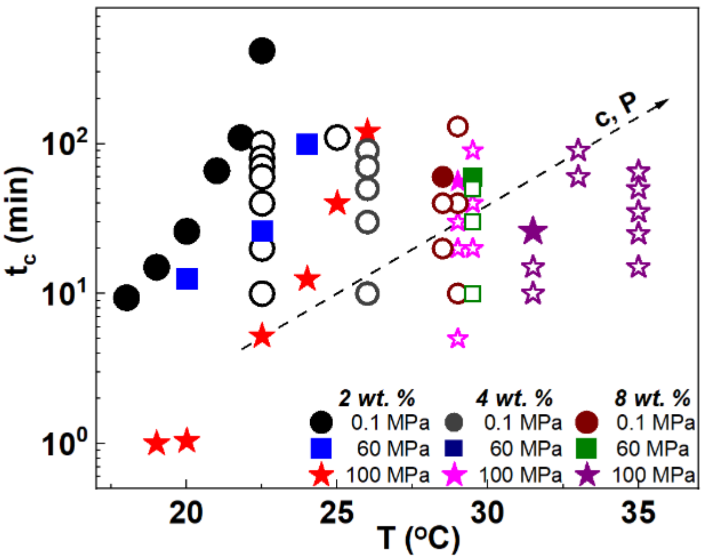

Figure S6. Gelation time (t_c) as a function of quench temperature at 0.1 (circles), 65 (squares) and 100 MPa (stars) at different gelatin concentrations, c = 2, 4 and 8 wt. %. Data extracted after Temperature jump (50 ºC to T_{ref}). Open symbols indicate formation of Sol; filled Gel formation.

iv. Sol viscosity

Analysis of DLS signals: To determine relaxation times from the $C(q,t)$, we analyzed the latter by means of inverse Laplace transformation (ILT), which was realized using the program CONTIN. The $C(q,t)$ is described as a superposition of exponentials, $\alpha C(q,t) = \int L(ln\tau)\exp(-t\tau)\, dln\tau$ where α is the amplitude of a relaxation mode and τ its characteristic relaxation time. This provides a continuous spectrum of relaxation times ($ln\tau$). The characteristic relaxation modes (in our case there is simply one mode with varying broadness) and their respective relaxation time(s) converted to intrinsic viscosity (-ies) through the use of Stokes – Einstein – Sutherland SES relation which, for the data presented are on Fig. 5 (B).

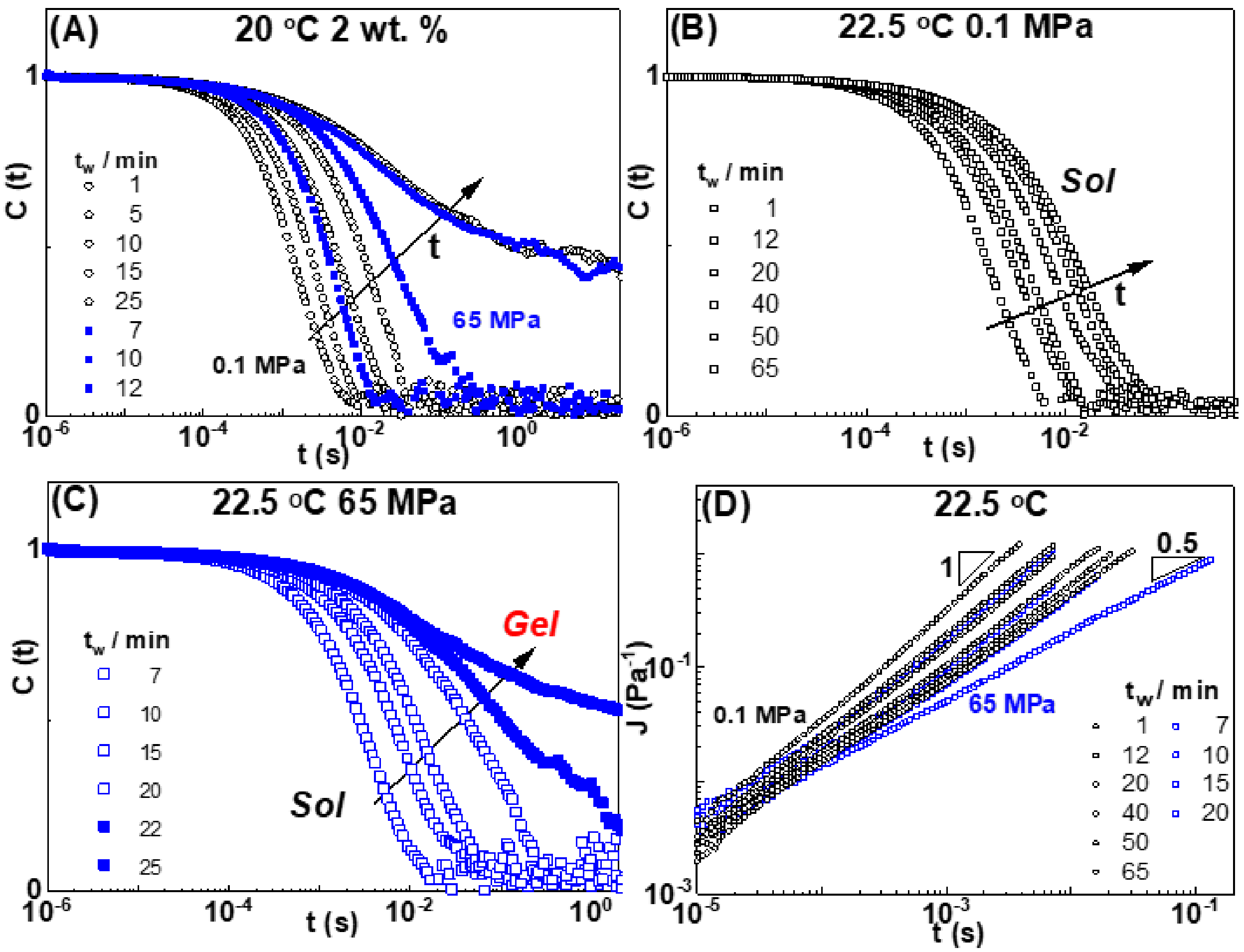

Figure S7. Time evolution Colloidal-probe DLS of Gelatin dispersions at c = 2 wt. % for different waiting times, t_w at: (A) 0.1 (open black spheres) and 65 MPa (filled blue squares) and 20 ^{O}C (B) 22.5 ºC, 0.1 MPa (C) 22.5 ºC, 65 MPa, (open squares attributed to sol phase, filled to gel) (D) respective creep compliance data derived from (B) (open black spheres) and (C) (open blue squares).

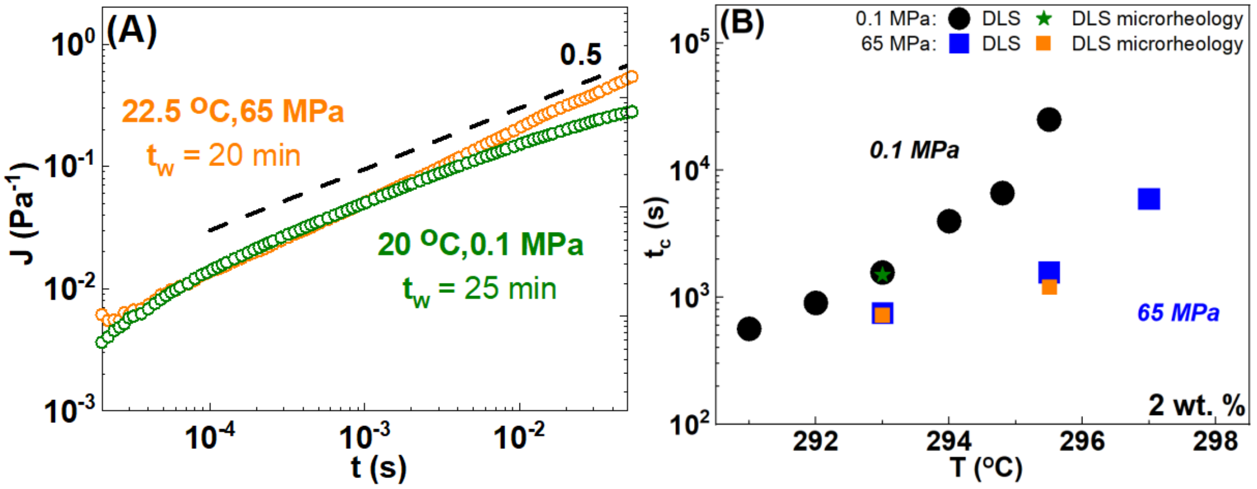

Figure S8. (A) Creep compliance of Gelatin dispersions at c = 2 wt. % for different waiting times, t_w and pressures at 22.5 ºC, 65 MPa, t_w = 20 minutes (orange open circles) and 20 ºC, 0.1 MPa, t_w = 25 minutes (green open circles). (B) Comparison of Critical gelation time (t_c) derived from DLS (black circles at 0.1 MPa, blue squares at 65 MPa) and DLS microrheology (green star at 0.1 MPa and orange square at 65 MPa) as a function of quench temperature at c = 2 wt.%.

v. Gel elasticity

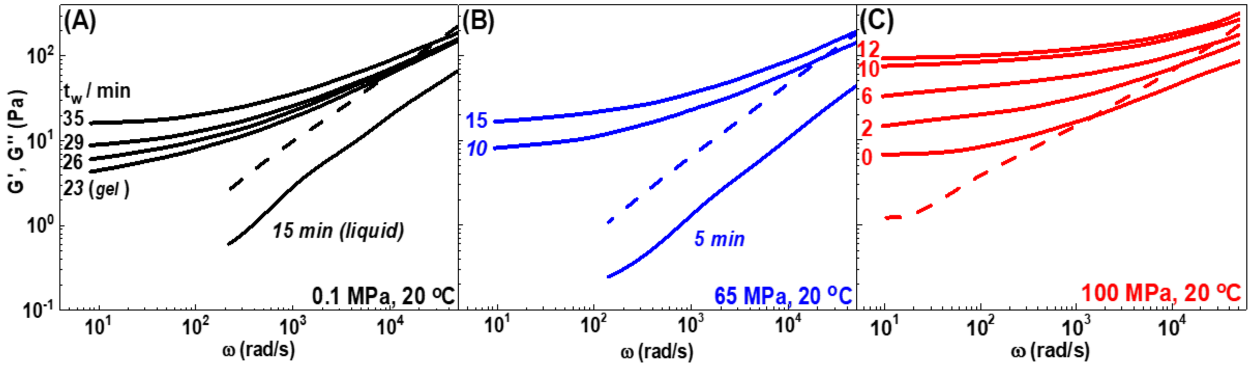

Figure S9. Time – Evolution, t_w of Storage (G', lines) and Loss (G'', dashed, lines) modulus (Pa) as a function of frequency, ω at (A) 0.1 (black lines), 65 (blue lines) and 100 MPa (red lines) of 2 wt. % gelatin solutions.

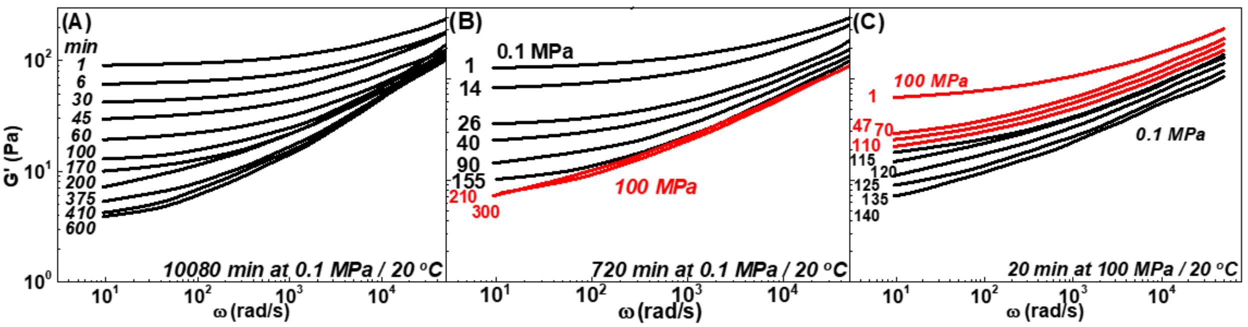

Figure S10. Time – Evolution, t_w of Storage (G', lines) and Loss (G'', dashed, lines) modulus (Pa) as a function of frequency, ω at 29 °C at 1 (black solid lines) and 100 MPa (red solid lines) after different gel preparation at (A) 10080 minutes at 0.1 MPa and 20 °C (B) 720 minutes at 0.1 MPa and 20 °C and (C) 20 min at 100 MPa and 20 °C.

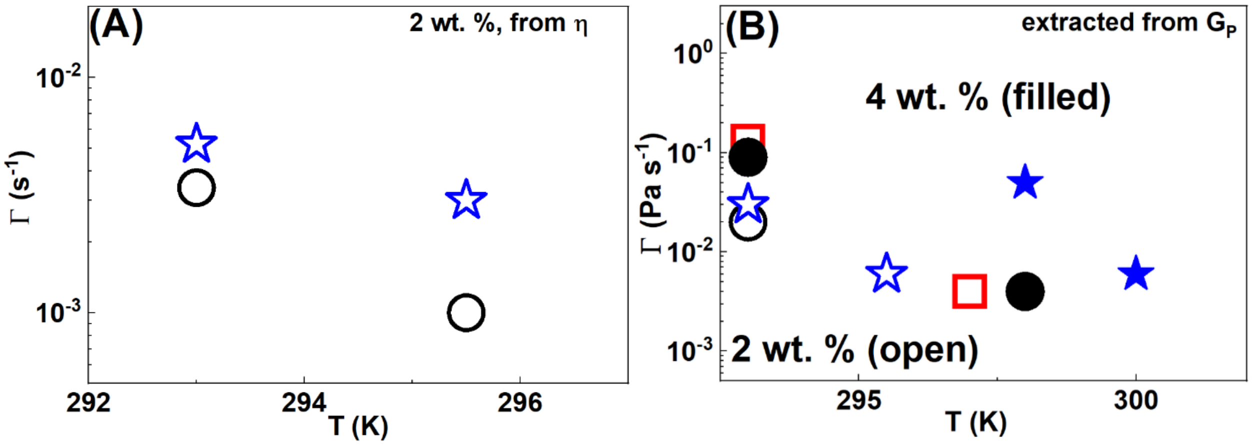

Figure S11. Rates versus temperature of gelatin dispersions extracted through (A) viscosity and (B) plateau modulus of figures 5B and 7C, respectively.

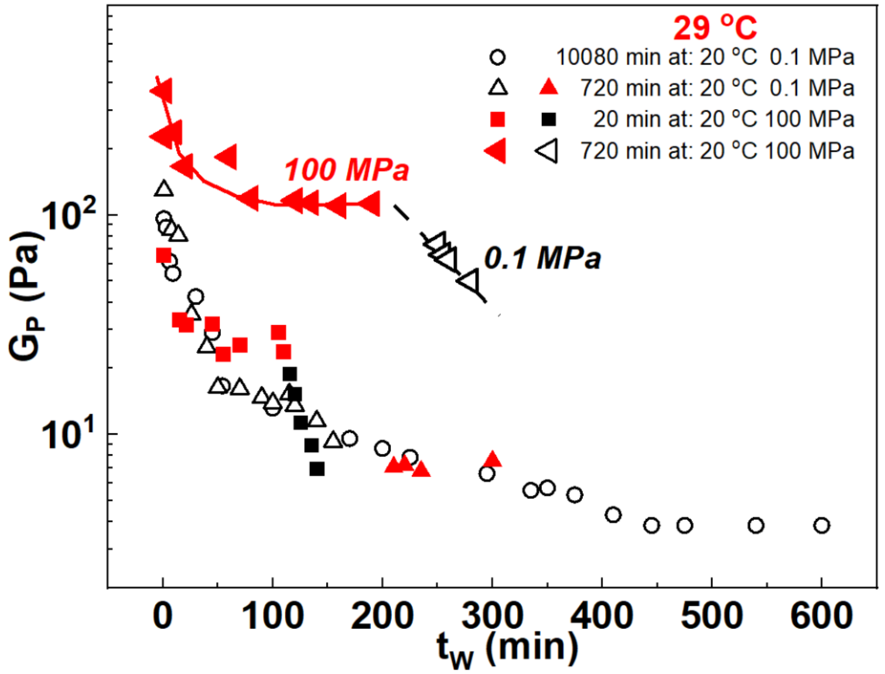

Figure S12. Evolution of plateau modulus $G_P(t)$ with waiting time, t_w at 29 ºC after different gel preparations for a 2 wt. % gelatin dispersion.
